# Metabolic Engineering of *Escherichia coli* for High-Level Production of (*R*)-Acetoin from Low-Cost Raw Materials

**DOI:** 10.3390/microorganisms11010203

**Published:** 2023-01-13

**Authors:** Mengxue Diao, Xianrui Chen, Jing Li, Ya’nan Shi, Bo Yu, Zhilin Ma, Jianxiu Li, Nengzhong Xie

**Affiliations:** 1 State Key Laboratory of Non-Food Biomass and Enzyme Technology, National Engineering Research Center for Non-Food Biorefinery, Guangxi Biomass Engineering Technology Research Center, Guangxi Academy of Sciences, 98 Daling Road, Nanning 530007, China; 2 State Key Laboratory for Conservation and Utilization of Subtropical Agro-Bioresources, Life Science and Technology College, Guangxi University, Nanning 530004, China; 3 CAS Key Laboratory of Microbial Physiological & Metabolic Engineering, Institute of Microbiology, Chinese Academy of Sciences, Beijing 100101, China

**Keywords:** (*R*)-acetoin, acetoin-tolerance, synthetic biology, low-cost feedstock

## Abstract

Acetoin is an important four-carbon platform chemical with versatile applications. Optically pure (*R*)-acetoin is more valuable than the racemate as it can be applied in the asymmetric synthesis of optically active α-hydroxy ketone derivatives, pharmaceuticals, and liquid crystal composites. As a cytotoxic solvent, acetoin at high concentrations severely limits culture performance and impedes the acetoin yield of cell factories. In this study, putative genes that may improve the resistance to acetoin for *Escherichia coli* were screened. To obtain a high-producing strain, the identified acetoin-resistance gene was overexpressed, and the synthetic pathway of (*R*)-acetoin was strengthened by optimizing the copy number of the key genes. The engineered *E*. *coli* strain GXASR-49RSF produced 81.62 g/L (*R*)-acetoin with an enantiomeric purity of 96.5% in the fed-batch fermentation using non-food raw materials in a 3-L fermenter. Combining the systematic approach developed in this study with the use of low-cost feedstock showed great potential for (*R*)-acetoin production via this cost-effective biotechnological process.

## 1. Introduction

Acetoin (3-hydroxy-2-butanone), the smallest natural chiral α-hydroxy ketone, is a high-value-added bio-based platform chemical with versatile applications [1,2], and is identified as one of the top 30 potential building blocks [3,4]. As a popular flavoring agent used in foods and cosmetics industries, due to its pleasant aroma, acetoin is recognized as safe (GRAS) by JECFA and US FDA [5]. Additionally, acetoin is also an important precursor of tetramethylpyrazine, an active pharmaceutical ingredient originally isolated from *Ligusticum wallichii* for curing cardiovascular and cerebrovascular diseases [6,7]. Acetoin has one chiral center in the molecule and, therefore, exists as two stereoisomers, (*R*)-acetoin and (*S*)-acetoin. Optically pure (*R*)-acetoin is more valuable than the racemate as it can be applied in the asymmetric synthesis of optically active α-hydroxy ketone derivatives, pharmaceuticals, and liquid crystal composites [3,8,9].

With the rapid development of synthetic biology, various microorganisms have been genetically engineered for producing (*R*)-acetoin [2,6,10,11,12,13]. However, requirements for complex nutrients and sugar substrates from many heterologous hosts increase both the production cost and process complexity. *Escherichia coli* is the most widely used host for (*R*)-acetoin production due to its low nutrition requirements, fast and high-density cultivation, clear genetic background, and genetic tractability [14]. Moreover, *E*. *coli* can use both pentose and hexose sugars from lignocellulose, as well as other inexpensive substrates such as seaweed hydrolysate and cellodextrin, which is economically attractive [15]. Thus, *E*. *coli* is an ideal candidate for (*R*)-acetoin production.

Many research groups have reported the fermentative production of (*R*)-acetoin by engineered *E*. *coli* [8,16]. For example, an engineered *E*. *coli* was constructed by introducing the *budRAB* genes and NADH oxidase gene, and the final recombinant strain produced 60.3 g/L (*R*)-acetoin in a 5-L bioreactor [17]. Xu and colleagues developed an engineered *E*. *coli* strain BL15 to efficiently produce acetoin from glucose. Under optimal conditions, 68.4 g/L acetoin was achieved by fed-batch fermentation [18]. In addition to the fermentative method, whole-cell catalysis of recombinant *E*. *coli* has also been developed to produce (*R*)-acetoin. Guo et al. constructed a recombinant *E*. *coli* by co-expressing (2*R*,3*R*)-2,3-butanediol dehydrogenase, NADH oxidase, and *Vitreoscilla* hemoglobin to achieve a high yield [19]. The whole-cell biocatalyst produced 86.74 g/L (*R*)-acetoin with a productivity of 3.61 g/L/h and a stereoisomeric purity of 97.89% from 93.73 g/L *meso*-2,3-butanediol. This result represents the highest reported concentration of (*R*)-acetoin achieved in *E*. *coli* to date. However, the high cost of *meso*-2,3-butanediol significantly increases the production cost and limits its use in industrial-scale applications.

Most studies on the fermentative production of (*R*)-acetoin have employed commercially pure sugars and yeast extract as feedstocks. Compared with using low-cost raw materials, relatively high concentrations of (*R*)-acetoin can be produced from glucose and yeast extract, but refined feedstocks are not economically feasible in industrial fermentation processes due to the high cost. Therefore, developing a fermentative method based on low-cost raw materials is crucial for the cost-competitive production of (*R*)-acetoin. Cassava (*Manihot esculenta*) is one of the most efficient crops in terms of starch production, and it is particularly tolerant to nutrient-poor or depleted soils in which the yields of other crops are very low [20]. This cheap and abundant crop is the basis of many fermented products such as ethanol and L-lactic acid [21,22,23]. However, little attention was paid to the fermentative production of (*R*)-acetoin using cassava. On the other hand, among various nitrogen sources, yeast extract is the best choice for both microbial growth and (*R*)-acetoin production and is most widely used in previous studies. For example, yeast extract was found to be the largest contributor to lactic acid production, and it accounted for about 38% of the total medium cost in the economic analysis [24]. However, the unit price of industrial yeast extract is estimated to be about 3.5 USD/kg (https://www.procurementresource.com/resource-center/yeast-extract-price-trends, accessed on 27 December 2022), which increases the medium cost and is economically unviable in industrial-scale fermentation. Cotton is one of the most important crops in the world, whereas cottonseed is considered an agricultural waste after cotton processing [25]. This abundant raw material offers promise as a low-cost nitrogen source for (*R*)-acetoin production. In the present study, we used cassava powder and cottonseed meal as feedstocks for the microbial production of (*R*)-acetoin.

Additionally, the cytotoxicity caused by excess acetoin negatively affects cell growth, which in turn limits the final concentration of acetoin in microbial cell factories [26]. It is therefore important to improve the acetoin tolerance of host cells in the context of large-scale biomanufacturing of acetoin [1,6,27]. With the development of synthetic biology in recent years, investigations on acetoin tolerance mechanisms and the introduction of relevant stress-resistant factors to construct stress-tolerant strains provide new strategies for improving acetoin production. In contrast to the traditional methods (e.g., adaptation and physical/chemical mutagenesis), introducing or modifying certain stress-resistant factors by molecular biological modifications, which avoids cumbersome experimental steps and long experimental periods, is more direct and efficient in improving microbial resistance. Because the cell membrane is the first barrier to the external environment, researchers have reported a number of strategies regarding alteration in membrane fluidity for enhancing tolerance to organic solvents in microbes. For example, tolerance mechanisms of *yghW* and *yibT* genes have been elucidated that the down-regulation of these two genes could dramatically improve the *n*-butanol tolerance of WT *E*. *coli* by affecting membrane fatty acid composition [28]. By overexpression of the peptidoglycan biosynthesis gene *murA2* from *Lactobacillus plantarum*, the tolerance of *E*. *coli* to organic solvents, such as ethanol, *n*-butanol and *i*-butanol, and ethanol production in cultures can be significantly improved [29]. The ethanol resistance and thermotolerance of fission yeast were enhanced by expressing an ergosterol biosynthesis enzyme from an edible mushroom due to alteration in membrane sterol and fatty acid composition [30]. In another aspect, Fisher and co-workers provided a promising solution by engineering efflux pumps to pump the inhibitory *n*-butanol out of the *E*. *coli* cell [31]. They generated variants of *E*. *coli* AcrB by directed evolution strategy and enhanced the growth rate by up to 25%.

In this study, we aimed to explore the acetoin tolerance and improve acetoin production in engineered *E*. *coli* using a combinatorial engineering strategy, including tolerance engineering and metabolic engineering. Currently, microbial tolerance to various organic solvents has been reported; however, to our knowledge, there are no reports regarding (*R*)-acetoin stress. Herein, we investigated the roles of putative acetoin-tolerant genes in the engineered *E*. *coli* and enhanced metabolic flux towards acetoin production by optimizing the copy number of the key genes. This systematic approach and the comprehensive utilization of low-cost feedstocks in this study could provide reference and guidance for developing cost-effective biotechnological processes for (*R*)-acetoin production.

## 2. Materials and Methods

### 2.1. Chemicals and Reagents

Acetoin (>98%) was purchased from Tokyo Chemical Industry Co., Ltd. (Shanghai, China). Acetonitrile (HPLC grade) was obtained from Sinopharm Chemical Reagent (Shanghai, China). Cassava powder and cottonseed meal were respectively obtained from Guangxi State Farms Mingyang Biochemical Group, Inc. (Nanning, China) and Jinan Oumi Biotechnology Co., Ltd. (Jinan, China). Thermostable α-amylase and glucoamylase obtained from Novozymes (Novozymes China Biotechnology Co., Ltd., Tianjin, China) were used for liquefaction and saccharification of raw starch as previously described [7]. The acid protease was purchased from Imperial Jade Biotechnology Co., Ltd. (Yinchuan, China) for protein hydrolysis [7].

### 2.2. Strains and Plasmids

All strains and plasmids used in this study are described in Table 1. *E*. *coli* DH5α was used for cloning and plasmid construction. *E*. *coli* MG1655 was engineered for acetoin production. To reduce by-products, strain GXASR-48 was derived from *E*. *coli* MG1655 by stepwise deletions of genes (*gldA*, *frdABCD*, *ackA-pta,* and *poxB*) via homologous recombination. Strain GXASR-49 was derived from GXASR-48 by inserting gene *tsf* at *yibT*. pTrc99A was used as the expression vector. The codon-optimized genes, including *budB* (encoding α-acetolactate synthase) and *budA* (encoding α-acetolactate decarboxylase) from *Enterobacter cloacae*, and *noxE* (encoding NADH oxidase) from *Lactobacillus brevis*, were used to construct plasmids pTrc99A-*budB*-*budA*-*noxE* or pRSF-*budB*-*budA*-*noxE*. Plasmid pRSF-*budB*-*budA*-*noxE* was constructed by replacing the *ori* of pTrc99A-*budB*-*budA*-*noxE* with the high-copy replicon *RSF* of pRSFDuet using a seamless cloning method. The recombinant plasmids were transformed into mutant *E*. *coli* strains for acetoin biosynthesis as well as for the balance of NADH/NAD^+^ redox. Primers were synthesized by GenSys Biotechnology Co., Ltd. (Nanning, China) and listed in Table S1.

### 2.3. Culture Conditions

Cells were routinely cultivated in Luria-Bertani (LB) medium. The composition of the initial fermentation medium (IFM) was as follows: 100 g/L glucose, 10 g/L peptone, 7 g/L yeast extract, 0.5 g/L NaCl, 0.2 g/L MgSO_4_, 0.5 g/L betaine monohydrate and 0.1 g/L thiamine. The cassava powder and cottonseed meal were enzymatically hydrolyzed as described previously to make the hydrolysate medium [7]. Briefly, 18.8 and 6.7% (*w/v*) cassava powder and cottonseed meal (pH 6.3) were pretreated by autoclaving with 10 mL of α-amylase solution (0.5 g CaCl_2_ was added to 4 mL α-amylase and diluted with distilled water to a total volume of 100 mL) at 121 °C for 15 min. Then, the autoclaved mixture was liquefied at 95 °C for 1 h with 15 mL of α-amylase solution in a water bath shaker (160 rpm). Finally, the liquefied mash was cooled to room temperature, adjusted to pH 4.3 with H_2_SO_4_, and saccharified at 55 °C for 24 h using 25 mL of 6% (*v/v*) glucoamylase and 0.6 g acid protease on a water bath shaker (160 rpm). After hydrolysis, the supernatant was collected by centrifugation at 6000 rpm for 5 min. Media for recombination strains were supplemented with 0.1 g/L ampicillin.

### 2.4. Screening of Acetoin Resistance Genes

Four putative organic-solvent-tolerant genes, including *gro*ESL (chaperonin), *mms*B (encoding 3-hydroxyisobutyrate dehydrogenase), *tsf* (protein chain elongation factor EF-Ts), and *PSEEN0851* (isochorismatase superfamily hydrolase), were first obtained through genome amplification or gene synthesis and then cloned into plasmid pTrc99A driven by IPTG inducible promoter P_trc_. The recombinant plasmids pTrc99A-*gro*ESL, pTrc99A-*mms*B, pTrc99A-*tsf,* and pTrc99A-*PSEEN0851* were respectively transformed to host GXASR-48 to investigate their acetoin resistance effects. We compared the growth of recombinant strains under the stress of acetoin at 45 or 50 g/L. GXASR-48 harboring the empty plasmid pTrc99A was used as control (CK).

For gene knocking-out of *yibT* or *yghW* in GXASR-48, CRISPR/Cas9 system was used as described previously [32]. The shooting sequences consisting of 500 bp upstream and downstream of the target gene were assembled by overlapping PCR. The selected sgRNA was cloned into plasmid pTarget, which was then electro-transformed into GXASR-48 accompanied by its shooting sequence. Then, we obtained correct deletion mutants by colony PCR and sequencing for further acetoin resistance experiments. The strain GXASR-48 was set as control.

### 2.5. Overexpression of Gene tsf

GXASR-49 (*E*. *coli* MG1655 Δ*gldA* Δ*frdABCD* Δ*ackA-pta* Δ*poxB* y*ibT*::*tsf*) was obtained by the deletion of *yibT* gene and integration of a single copy of *tsf* gene in *E*. *coli* by CRISPR/Cas9 gene editing. CRISPR RNAs were designed using online gene editing tools (http://crispr.tefor.net/, accessed on 9 September 2022) and listed in Table S1. The plasmid skeleton of pTarget-tdcC without *N20* site was amplified with pTarget-F/R primers. The *N20* and purified skeleton of pTarget were assembled by Minerva Super Fusion Cloning Kit (US Everbright^®^ Inc., Suzhou, China) following the manufacturer’s instructions. The recombinant plasmid pTarget-*N20* was transformed into *E*. *coli* DH5α. Positive colonies were verified by sequencing. Gene *tsf* and the upstream and downstream homologous arms of *yibT* were amplified from genomic DNA of *E*. *coli* MG1655 with primers *tsf*-F/R, *yibt* (up)-F/R, and *yibt* (down)-F/R. The resultant strain was designated as GXASR-49.

### 2.6. Stress Effects of Acetoin on Strain GXASR-48p

The influence of acetoin supplementation (0, 20, and 40 g/L) on the growth of strain GXASR-48p was investigated in the LB medium. GXASR-48p was precultured in 5 mL LB medium for 8–12 h, and then inoculated (10%, *v/v*) into 40 mL LB medium in 250 mL flasks. After 4 h of incubation, different concentrations of acetoin were added to the culture. The samples (1 mL) were withdrawn at intervals to measure the optical density at 600 nm (OD_600_). All the experiments were carried out in triplicate unless otherwise stated.

### 2.7. Production of Acetoin in Shake Flasks

Shake-flask fermentation was conducted in 250 mL Erlenmeyer flasks containing 40 mL of IFM (pH 6.5) using strains GXASR-48p and GXASR-49p. The preculture was inoculated (10%, *v/v*) into the medium and performed on a rotary shaker at 250 rpm and 37 °C. To examine the fermentation performance of strains GXASR-49RSF and GXASR-49p, the batch fermentations in shake flasks were carried out in 500 mL Erlenmeyer flasks containing 80 mL IFM or hydrolysate medium with an inoculation proportion of 10% at 250 rpm and 37 °C. To compare the fermentation performance of the different strains, samples were collected at the designated time points and subjected to the measurements of OD_600_, the concentration of residual glucose, and productions of acetoin and the major by-product 2,3-butanediol (see Section 2.9).

### 2.8. Fed-Batch Fermentation in a 3-L Fermenter

Fed-batch fermentation to produce acetoin was carried out in a 3-L fermenter containing 1.5 L hydrolysate medium using strains GXASR-48p and GXASR-49p with pH controlled at 6.5 using 50% (*v/v*) ammonia solution. The inoculation size was 10% (*v/v*). The initial concentration of glucose for fermentation was about 110 g/L. The feeding strategy was as follows: when residual glucose from hydrolyzed cassava powder decreased below 40 g/L, the concentrated hydrolysate was added into the fermentation broth until the glucose concentration reached approximately 100 g/L; when the residual glucose again dropped to around 60 g/L, the hydrolysate was supplemented to reach a glucose concentration of 100 g/L. The initial agitation speed was 400 rpm and increased to 500 rpm after the first fed-batch cycle. The temperature and airflow were maintained at 37 °C and 1.5 vvm, respectively. Samples were taken at intervals to measure the OD_600_, residual glucose, and concentrations of acetoin and 2,3-butanediol.

The fermentation strategy using GXASR-49RSF was slightly modified on the basis of the strategy for strains GXASR-48p and GXASR-49p as follows: the initial glucose concentration hydrolyzed from raw material was about 110 g/L, and the concentrated hydrolysate was supplemented to make the glucose concentration to around 100 g/L when residual glucose dropped below 40 g/L; when the residual glucose decreased to 20 g/L, the concentrated hydrolysate was added to make the glucose concentration to about 60 g/L. The initial agitation speed was 400 rpm during the fermentation course. Other fermentation parameters were unchanged.

### 2.9. Analytical Methods

Cell growth was monitored by measuring OD_600_ using a UV-VIS spectrometer (UV-1900i, Shimadzu, Suzhou, China). The supernatant of fermentation broth was obtained by centrifugation at 12,500 rpm for 3 min and the residual glucose was quantified with a biosensor analyzer (SBA-40D, Biology Institute of Shandong Academy of Sciences, Jinan, China). Quantifications of acetoin and 2,3-butanediol in the filtered supernatant (0.22-µm nylon filter) were carried out using gas chromatography (GC). The GC-FID analysis was performed with an Agilent 7890A (Agilent, Santa Clara, CA, USA) equipped with a Phenomenex ZB-WAX Plus column (30 m × 0.32 mm × 0.25 µm) using high-purity nitrogen as carrier gas at a flow rate of 1.6 mL/min. The oven temperature program was as follows: 100 °C for 1 min, 20 °C/min ramp to 180 °C, and held at 180 °C for 3 min. The injector and detector temperatures were 250 °C. The optical purity of (*R*)-acetoin was determined as described in our previous study [7]. In brief, (*R*)- and (*S*)-acetoin in the fermentation broth were extracted with ethyl acetate and quantified by an Agilent GC system (7890A) using an Agilent CycloSil-B chiral column (0.32 mm × 0.25 mm × 30 m). The optical purity of (*R*)-acetoin was calculated as follows: (*R*)/[(*S*) + (*R*)] × 100%, where (*R*) and (*S*) stand for the concentrations of the two stereoisomers. Concentrations of formate, acetate, and succinate were detected by high-performance liquid chromatography (HPLC) (Dionex UltiMate 3000 system, Saint Louis, MO, USA) using a Carbomix H-NP 10 column (Sepax, Newark, NJ, USA, 7.8 × 300 mm). The organic acid by-products were analyzed by UV 210 nm, column temperature 55 °C, and 2.5 mM H_2_SO_4_ as mobile phase with 0.6 mL/min flow rate.

## 3. Results and Discussion

### 3.1. Optimization of Metabolic Pathways in Acetoin-Producting Strains

Previously, the biosynthesis pathway of acetoin was constructed in *E*. *coli* MG1655 by our group [7]. However, a high content of organic acid by-products, such as formate, acetate, and succinate, was produced by this engineered strain *E*. *coli* MG1655p (Table 2). Glycerol can be a carbon source for the biosynthesis of organic acids. Lv et al. knocked out *gld*A encoding glycerol dehydrogenase in an engineered acetoin-producing strain, *Serratia marcescens,* to reduce the synthesis of by-products downstream of glycerol [33]. Hao et al. realized the reduction of succinate by 37.4% by knocking out fumarate reductase *frdB* [34]. Under anaerobic conditions, pyruvate forms acetyl-CoA, which was converted into acetate by phosphotransacetylase (PTA) and acetate kinase (ACKA). In addition, pyruvate can form acetate by pyruvate oxidase (POXB). Therefore, the accumulation of acetate could be reduced by knocking out *ackA*-*pta* and *poxB* [35,36].

To further improve the metabolic pathway for acetoin production, *E*. *coli* MG1655 was used as the starting strain for the deletion of *gldA*, *frdABCD*, *poxB*, *ackA,* and *pta*. The resulting strain, GXASR-48, was transformed with plasmid pTrc99A-*budB*-*budA*-*noxE* to obtain the recombinant strain GXASR-48p (Table 1). After fermentation in 250 mL shake flasks containing 40 mL of IFM using GXASR-48p, the contents of formate, acetate, and succinate in the fermentation broth respectively decreased by 100%, 69.95%, and 89.58% compared with that produced by *E*. *coli* MG1655p. When glucose is the main carbon source, it is metabolized into dihydroxyacetone phosphate (DHAP). DHAP is catalyzed by the glycerol dehydrogenase encoded by the *gldA* gene to produce glycerol, which serves as a substrate for the synthesis of organic acids. The result indicated that the accumulation of acidic by-products during fermentation could be effectively alleviated by limiting the downstream synthesis pathway of organic acids from glycerol and pyruvate via metabolic engineering approaches. However, acetoin production of GXASR-48p (30.87 g/L) was lower than that of *E. coli* MG1655p (32.70 g/L). This was because deletions of *frdABCD*, *gldA*, *poxB*, *ackA,* and *pta* might break the rigidity and balance of the cellular metabolic network, and the normal physiological metabolic activities of cells were affected to some extent.

### 3.2. Evaluation of Acetoin Toxicity on Engineered Strain GXASR-48p

**Figure 1:**
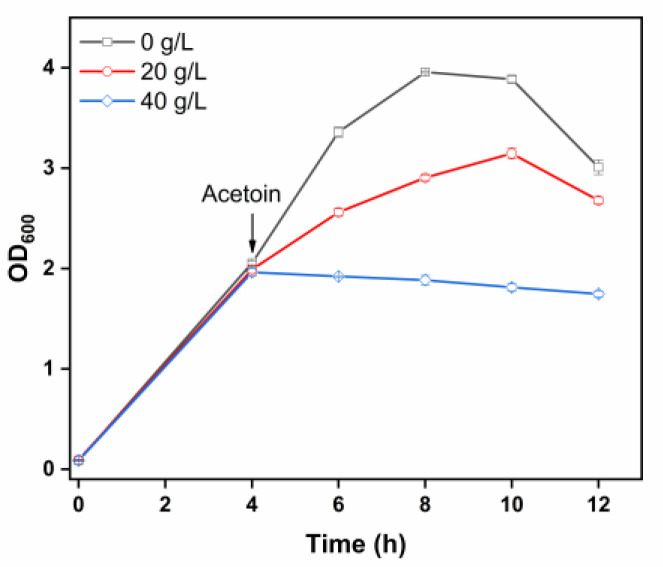
Effect of acetoin on the cell growth of strain GXASR-48p in LB medium. Error bars represent the standard deviation (*n* = 3). Due to small variability, some error bars hidden behind the symbols are not apparent.

Strain GXASR-48 was derived from *E*. *coli* MG1655 by deletion of *gldA*, *frdABCD*, *ackA*-*pta,* and *poxB*, and the recombinant plasmid pTrc99A-*budB*-*budA*-*noxE* was transformed into strain GXASR-48, resulting in strain GXASR-48p. To investigate the toxic effects of acetoin on cell physiology, we analyzed the tolerance of strain GXASR-48p to various acetoin concentrations (0, 20, and 40 g/L), as shown in Figure 1. Without the addition of acetoin, the maximal OD_600_ reached 4.0 at 8 h. In contrast, the cell growth was largely restrained with the addition of 20 g/L acetoin, resulting in a maximal OD_600_ of 3.16 at 10 h. When the concentration of acetoin increased to 40 g/L, the cell growth was significantly inhibited, and the OD_600_ gradually decreased from 4 h. The result demonstrates that acetoin at high levels results in toxicity to the *E*. *coli* cells. As previously reported, acetoin can affect cell viability by damaging DNA and proteins, as well as altering the membrane lipid composition of prokaryotic and eukaryotic cells [26]. As a result, there is an urgent need to enhance the tolerance of engineered strains to acetoin at toxic concentrations.

### 3.3. Screen of Acetoin Resistance Genes

**Figure 2:**
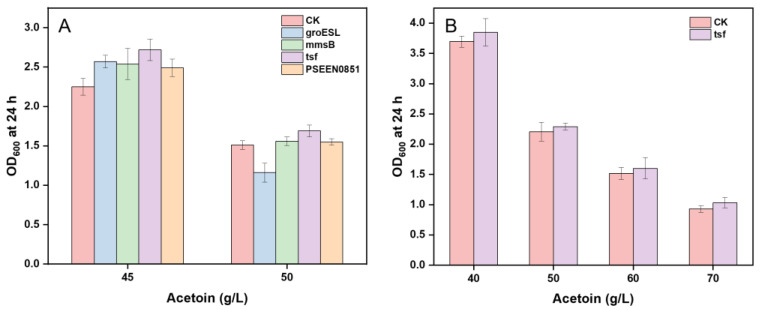
(**A**) Screening of putative acetoin-tolerant genes with stress of 45 or 50 g/L acetoin in GXASR-48 by overexpressing individually in plasmid pTrc99A, CK: GXASR-48-CK, *gro*ESL: GXASR-48-groESL, *mms*B: GXASR-48-mmsB, *tsf*: GXASR-48-tsf, *PSEEN0851*: GXASR-48-PSEEN0851. (**B**) Further identifying the acetoin resistance of GXASR-48-tsf with a gradient concentration of acetoin. Error bars represent the standard deviation (*n* = 3).

Bacterial tolerance to metabolites is still a limiting factor in the biosynthesis of toxic chemicals. To date, there were few reports on the acetoin resistance mechanism of bacteria, except for some studies on the tolerance of *E*. *coli* to alcohols and alkanes. Zingaro et al. [37] found heterologous expression of the GroESL chaperone system with its natural promoter in *E*. *coli* significantly improved its tolerance to several toxic alcohols. Ni et al. [38] identified the effect of *mms*B (encoding 3-hydroxyisobutyrate dehydrogenase), *tsf* (protein chain elongation factor EF-Ts), and *PSEEN0851* (isochorismatase superfamily hydrolase) on increasing resistance to several organic solvents such as *n*-butanol when heterologously expressed in *E*. *coli*. Given the similarity of molecular structures between *n*-butanol and acetoin, we tested the growth of strain GXASR-48 harboring the above genes in recombinant plasmids, pTrc99A-*gro*ESL, pTrc99A-*mms*B, pTrc99A-*tsf,* and pTrc99A-*PSEEN0851*, under the stress of acetoin at 45 or 50 g/L, respectively. The results showed that overexpression of *tsf* increased the growth of GXASR-48 by 20.9% and 11.9% under 45 and 50 g/L acetoin stress, respectively, which was the most effective among the four putative organic-solvent-tolerant genes compared to the control strain (Figure 2A). Moreover, the subsequently identified results with a gradient acetoin concentration of 40–70 g/L showed that GXASR-48-tsf did have growth advantages (Figure 2B). This was because gene *tsf* might help to increase the expression level of some stress response proteins and improve the tolerance to acetoin [38].

**Figure 3:**
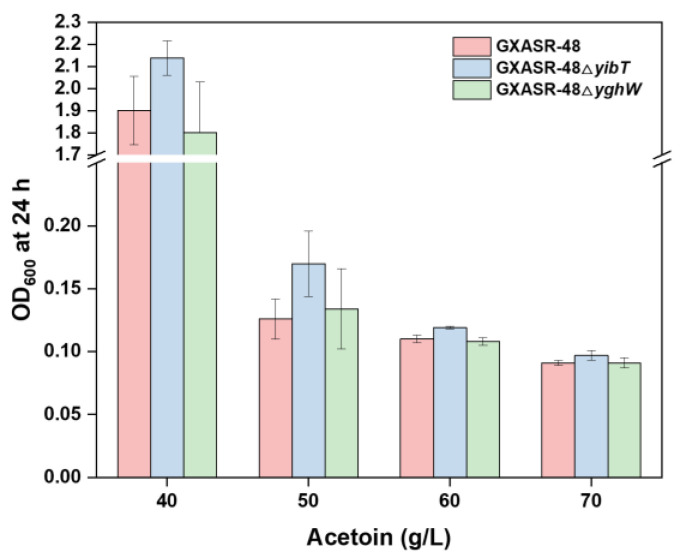
The effect of knocking-out of *yibT* or *yghW* individually in GXASR-48 on acetoin resistance. The concentration of exogenously added acetoin varied from 40 to 70 g/L. Error bars represent the standard deviation (*n* = 3).

Acetoin is an organic solvent that can change the potential and permeability of cell membranes, causing the leakage of intracellular proteins. Therefore, cell membranes may be a key factor for microorganisms to resist acetoin. It was reported that expression of cis-trans isomerase (*Cti*) from *Pseudomonas aeruginosa* reduced the membrane fluidity of *E*. *coli* MG1655, whose resistance to exogenously added octanoic acid was significantly increased [39]. Gong et al. also found the average membrane lipid acyl chain length of *E*. *coli* was increased by overexpressing *S*-malonyltransferase *fab*D (acyl-carrier-protein), and the engineered strain had improved growth towards *n*-butanol stress [40]. In a previous report, two down-regulated genes, *yibT* and *yghW*, were explored through transcriptome profile analysis in the 2% (*v/v*) butanol-tolerant *E*. *coli* B8 [28]. It was found that these genes affect butanol resistance by regulating the fatty acid composition in the cell membrane. Hence, we tested whether these genes could improve the tolerance of *E*. *coli* to acetoin by gene knocking out. The result showed that the engineered strain GXASR-48ΔyibT exhibited stronger acetoin resistance, whose growth improved by 12.5% and 34.9%, respectively, when exposed to 40 and 50 g/L acetoin (Figure 3). Gene *yibT* is a membrane-related gene, and knocking out *yibT* would represent a higher surface hydrophobicity of the cytomembrane [28]. Thus, the cell surface is less permeable, which helps prevent the invasion of toxic compounds such as acetoin. Given the above positive results, the strain GXASR-49 was further constructed by inserting *tsf* into the gene site of *yibT*, with synchronous deletion of the *yibT* gene to enhance the tolerant capacity to acetoin.

### 3.4. Effect of Resistant Gene tsf on Acetoin Production

**Figure 4:**
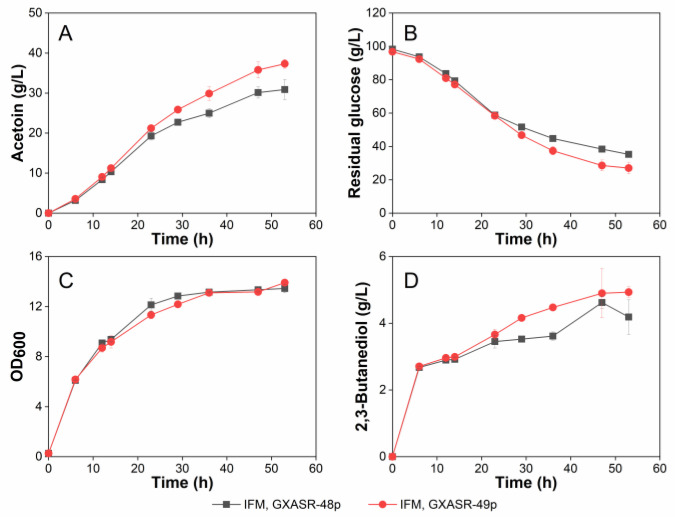
Batch fermentation using strains GXASR-48p and GXASR-49p in 250 mL shake flasks containing 40 mL of IFM. (**A**) Acetoin production; (**B**) glucose consumption; (**C**) time course profile of cell growth; (**D**) 2,3-Butanediol production. Error bars represent the standard deviation (*n* = 3). Due to small variability, some error bars hidden behind the symbols are not apparent.

The plasmid of pTrc99A-*budB*-*budA*-*noxE* was then transformed into GXASR-49, resulting in GXASR-49p. As shown in Figure 4D, the production of 2,3-butanediol using IFM remained at a relatively low level throughout the course of fermentation. The growth curves of strains GXASR-48p and GXASR-49p were similar before 47 h, but the OD_600_ of GXASR-49p (OD_600_ = 13.91) surpassed that of GXASR-48p (OD_600_ = 13.45) at 53 h and showed a continuously rising trend (Figure 4C). This time profile of OD_600_ might be due to the adaptation process of GXASR-49p to acetoin stress between 34 and 47 hr. After adapting to this stress, the strain continued to increase the biomass using the carbon source from residual glucose. On the contrary, the OD_600_ of GXASR-48p plateaued from 47 h. This result indicated that in the late fermentation stage, GXASR-49p maintained robust growth and exhibited better resistance to acetoin. Besides, strain GXASR-49p exhibited a faster glucose consumption along with a higher production of acetoin at 37.33 g/L, which was 20.92% higher than that of strain GXASR-48p. As seen in Figure 4, on one hand, GXASR-49p showed a higher capacity to grow, allowing a higher glucose consumption than that of GXASR-48p, which led to higher titers of acetoin and 2,3-butanediol. On the other hand, GXASR-49p maintained better cell viability and glucose consumption at a higher acetoin concentration, indicating that its tolerance to acetoin was indeed improved by inserting *tsf* at *yibT* in the genome.

**Figure 5:**
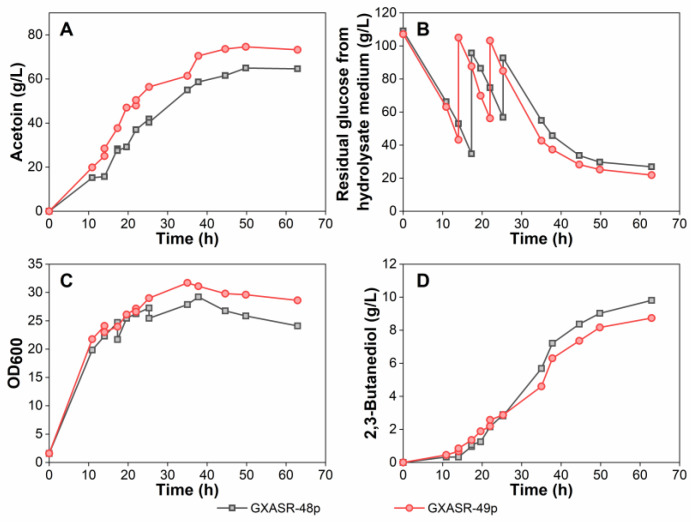
Fed-batch fermentation in a 3-L fermenter containing 1.5 L of hydrolysate medium using strains GXASR-48p and GXASR-49p. (**A**) Acetoin production; (**B**) glucose consumption; (**C**) time course profile of cell growth; (**D**) 2,3-Butanediol production.

To further improve the production of acetoin, fed-batch fermentation experiments were carried out in a 3-L fermenter with these two transformants using the hydrolysate medium. As seen in Figure 5, strain GXASR-49p reached a maximum acetoin titer of 74.61 g/L at 50 h, which was 14.8% higher than that of strain GXASR-48p. The acetoin production was 1.69 times higher than that of GXASR-49p in shake-flask fermentation using the hydrolysate medium (Figure 4A). Moreover, the OD_600_ of GXASR-49p was higher than that of GXASR-48p, and the differences in OD_600_ in the 3-L fermenter (Figure 5) were also more obvious than that in shake flasks (Figure 4). This indicated that inserting the *tsf* gene at the *yibT* site was beneficial for strains to maintain better growth in a higher concentration of acetoin, i.e., overexpression of *tsf* and the knocking-out of *yibT* can improve the stress resistance to acetoin, and the resistance was more pronounced with the increase in acetoin concentrations. Overall, acetoin resistance and production of GXASR-49p were successfully improved by integrating a single copy *tsf* gene, encoding the elongation factor EF-Ts, at gene *yibT* for fatty acid synthesis in the GXASR-48 genome.

### 3.5. Effect of Altering the Expression Level of Pathway Genes on Acetoin Production

Plasmids are extra-chromosomal self-replicating genetic elements [41]. Generally, the higher the copy number of a plasmid, the higher the expression levels of the gene/protein of interest [42]. To investigate the potential of the high copy-number replicon, strain GXASR-49RSF was constructed by transforming pRSF-*budB*-*budA*-*noxE* into strain GXASR-49. The recombinant plasmid, termed pRSF-*budB*-*budA*-*noxE*, was derived from pTrc99A-*budB*-*budA*-*noxE* by replacing its replicon by RSF replicon of plasmid pRSFDuet via seamless cloning technique. Strains GXASR-49RSF and GXASR-49p were employed in batch fermentation or fed-batch fermentation in 500 mL shake flasks containing 80 mL of IFM.

**Figure 6:**
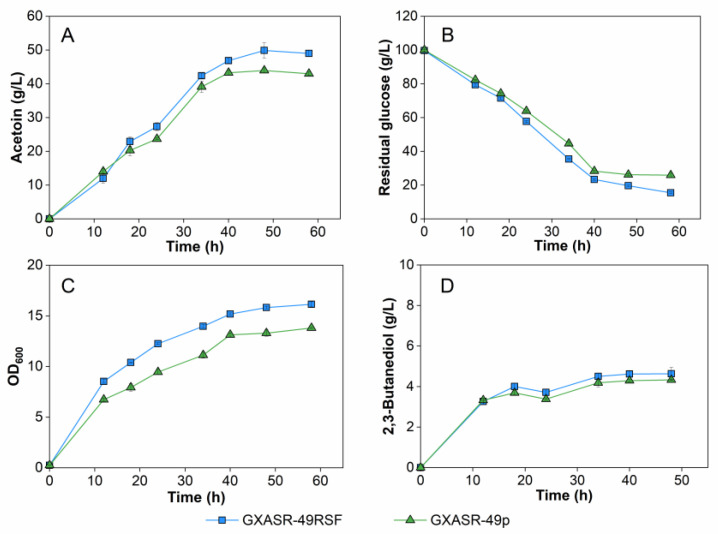
Batch fermentation by strains GXASR-49RSF and GXASR-49p in 500 mL shake flasks containing 80 mL of IFM medium. (**A**) Acetoin production during fermentation; (**B**) concentration of residual glucose in the fermentation broth; (**C**) time course profile of cell growth; (**D**) 2,3-Butanediol production during fermentation. Error bars represent the standard deviation (*n* = 3). Due to small variability, some error bars hidden behind the symbols are not apparent.

During the batch fermentation, the production of 2,3-butanediol of the two strains remained at a low level (Figure 6D). Strain GXASR-49RSF was advantageous in cell growth, glucose consumption, and acetoin production compared to GXASR-49p. As the fermentation approached the end, the highest production of acetoin attained 49.88 g/L, along with 15.47 g/L of residual glucose. Moreover, although GXASR-49RSF showed stronger growth than GXASR-49p, OD_600_ values of both strains continuously increased throughout the course of fermentation, reaching the maximal OD_600_ value of 16.14 and 13.81 at 58 h, respectively. In contrast, the OD_600_ of GXASR-48p plateaued around 13 since 36 h, while acetoin concentration was between 24.96 and 30.87 g/L (Figure 4). These results indicated that GXASR-49p and GXASR-49RSF had better tolerance at higher acetoin concentrations (> 40 g/L), which was possibly attributed to the insertion of *tsf* at *yibT*. Glucose consumption and acetoin production of GXASR-49RSF were 14.09 and 13.54% higher than that of GXASR-49p, respectively, demonstrating that the metabolic capability of cells can be enhanced by replacing the original replicon with the RSF replicon of plasmid pRSFDuet.

**Figure 7:**
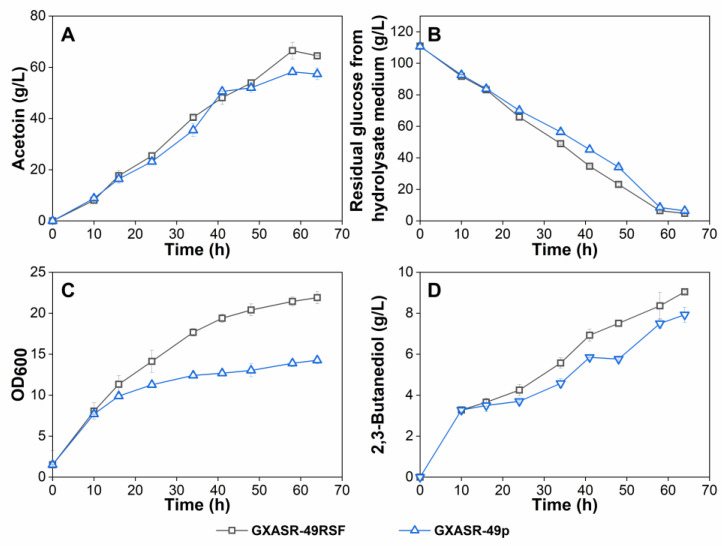
Batch fermentation using strains GXASR-49RSF and GXASR-49p in 500 mL shake flasks containing 80 mL of hydrolysate medium. (**A**) Time course profile of cell growth; (**B**) glucose consumption; (**C**) acetoin production; (**D**) 2,3-Butanediol production during fermentation. Error bars represent the standard deviation (*n* = 3). Due to small variability, some error bars hidden behind the symbols are not apparent.

Further shake-flask experiments were carried out in batch fermentation using GXASR-49p and GXASR-49RSF with the hydrolysate medium from low-cost cassava powder and cottonseed meal. As shown in Figure 7, the concentrations of by-product 2,3-butanediol produced by the two engineered strains in hydrolysate medium were slightly higher than that in IFM but remained at a low level (below 10 g/L). However, both strains produced higher acetoin in the hydrolysate medium. GXASR-49RSF showed better fermentation performance, generating 66.5 g/L acetoin in 60 h, which was 14.36% higher than that of GXASR-49p. In addition, GXASR-49RSF exhibited better cell growth in the mid- and late-fermentation phases, which was possibly due to the properly increased copy number of the recombinant plasmid. These results indicated again that replacing the replicon *ori* of pTrc99A-*budB*-*budA*-*noxE* with RSF replicon improved the production of acetoin in engineered *E*. *coli*. Moreover, the acetoin production, glucose consumption, and OD_600_ of both GXASR49RSF and GXASR49p recorded in the hydrolysate medium were all higher than that in IFM during batch or fed-batch fermentation, as shown in Figure 4 and Figure 5. This indicated that the hydrolysate medium is more beneficial to the engineered strain for acetoin production, due to richer nutrients and trace elements contained in the hydrolysates. This study aimed to achieve a higher acetoin production by improving the acetoin resistance of strains, which in fact improves cell growth. In another word, the higher the acetoin production, the more differences in stress resistance can be reflected.

**Figure 8:**
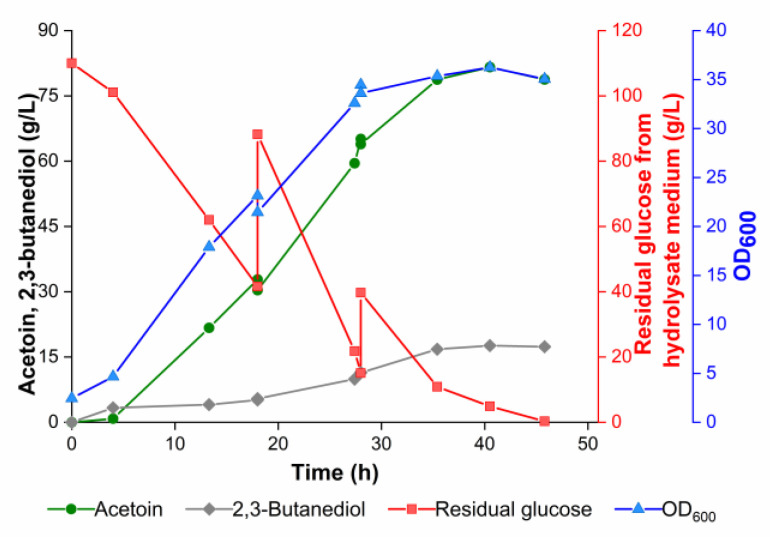
Fed-batch fermentation of acetoin in a 3-L fermenter containing 1.5 L of hydrolysate medium using strain GXASR-49RSF.

Due to the good performance of GXASR-49RSF in shake-flask fermentation, it was chosen for fed-batch fermentation in a 3-L fermenter using the hydrolysate medium. As shown in Figure 8, the glucose obtained from the hydrolysate of low-cost feedstocks was consumed at around 40 h, and the concentration of the by-product remained at a relatively low level throughout the course of fermentation with a maximum value of 17.51 g/L at 40 h. Due to an adequate afflux of substrate and nutrients from 0 to 30 h, GXASR-49RSF exhibited high metabolic viability and robust performance. After 40 h, the OD_600_ of culture broth began to decrease along with the consumption of glucose. Accordingly, the concentration of acetoin in the fermentation broth rapidly increased within the first 35 h of fermentation and then slowly increased till 40 h. The maximum concentration of acetoin reached 81.62 g/L, which increased by 9.4% compared to that produced by GXASR-49p (Figure 5), with high optical purity at 96.5%. Moreover, glucose was exhausted in the fermentation broth by the end of fermentation, which facilitates downstream processing. According to the market price (https://www.alibaba.com/?spm=a2700.7724857.scGlobalHomeHeader.4.157075abfwgPJr, accessed on 9 January 2023), cassava powder was about 300 USD/t, while the industrial grade glucose was about 400 USD/t, and cottonseed meal was about 1500 USD/t, while the industrial grade yeast extract or peptone was about 3500 USD/t and 2500 USD/t, respectively. Therefore, cassava powder and cottonseed meal used in this study provide a new approach for industrial acetoin fermentation production with cheap raw materials.

## 4. Conclusions

Acetoin at a high concentration is toxic to microorganisms and thus hinders the biomanufacturing of acetoin in microbial cell factories. In this work, we addressed this issue by genetic engineering to improve the tolerance of *E*. *coli* to acetoin, enhanced the metabolic capability of cells by increasing the expression level of key target genes and convert cheap feedstocks into value-added acetoin at a high level. Future studies will focus on more acetoin resistance elements that were underexplored, as well as the mechanisms of acetoin tolerance of microorganisms by using transcriptome analysis or other approaches. This work sheds light on the acetoin-resistance of engineered *E*. *coli* and has implications for future studies on the bioproduction of (*R*)-acetoin.

## Figures and Tables

**Table 1:** Strains and plasmids used in this study.

Strains or Plasmids	Descriptions	Sources
Strains		
*E*. *coli* MG1655	Host strain for heterologous biosynthesis of acetoin	Lab collection
*E*. *coli* MG1655p	*E*. *coli* MG1655 harboring pTrc99A-*budB*-*budA*-*noxE*	Lab collection
*E*. *coli* DH5a	Host strain for cloning	Lab collection
GXASR-48	*E*. *coli* MG1655 Δ*gldA* Δ*frdABCD* Δ*ackA-pta* Δ*poxB*	This study
GXASR-48-CK	GXASR-48 harboring pTrc99A	This study
GXASR-48-groESL	GXASR-48 harboring pTrc99A-*gro*ESL	This study
GXASR-48-mmsB	GXASR-48 harboring pTrc99A-*mms*B	This study
GXASR-48-tsf	GXASR-48 harboring pTrc99A-*tsf*	This study
GXASR-48-PSEEN0851	GXASR-48 harboring pTrc99A-*PSEEN0851*	This study
GXASR-48ΔyibT	*E*. *coli* MG1655 Δ*gldA* Δ*frdABCD* Δ*ackA-pta* Δ*poxB* Δ*yibT*	This study
GXASR-48ΔyghW	*E*. *coli* MG1655 Δ*gldA* Δ*frdABCD* Δ*ackA-pta* Δ*poxB* Δ*yghW*	This study
GXASR-49	*E*. *coli* MG1655 Δ*gldA* Δ*frdABCD* Δ*ackA-pta* Δ*poxB yibT*:: *tsf*	This study
GXASR-48p	GXASR-48 harboring pTrc99A-*budB*-*budA*-*noxE*	This study
GXASR-49p	GXASR-49 harboring pTrc99A-*budB*-*budA*-*noxE*	This study
GXASR-48RSF	GXASR-48 harboring pRSF-*budB*-*budA*-*noxE*	This study
GXASR-49RSF	GXASR-49 harboring pRSF-*budB*-*budA*-*noxE*	This study
Plasmids		
pTrc99A	Amp^R^	Lab collection
pTrc99A-*budB*-*budA*-*noxE*	Amp^R^	Lab collection
pTrc99A-*gro*ESL	Amp^R^	This study
pTrc99A-*mms*B	Amp^R^	This study
pTrc99A-*tsf*	Amp^R^	This study
pTrc99A-*PSEEN0851*	Amp^R^	This study
pRSFDuet	Kan^R^	Lab collection
pRSF-*budB*-*budA*-*noxE*	Replace the *ori* of pTrc99A with RSF replicon of pRSFDuet	This study
pTarget-*tdcC*	Str^R^ (constitutive expression)	Kindly provided by Prof. Bo Yu, Chinese Academy of Sciences
pCas	Kan^R^	

Amp^R^, ampicillin resistance; Kan^R^, kanamycin resistance.

**Table 2:** Acidic by-products of metabolic engineered strains.

Strains	Formate (g/L)	Acetate (g/L)	Succinate (g/L)
*E*. *coli* MG1655p	0.41 ± 0.05	2.13 ± 0.08	1.44 ± 0.19
GXASR-48p	ND ^a^	0.64 ± 0.10	0.15 ± 0.06

^a^ ND, not detected.

## Data Availability

Not applicable.

## Supplementary Materials

The following supporting information can be downloaded at: https://www.mdpi.com/article/10.3390/microorganisms11010203/s1, Figure S1: GC spectrum of fermentation broth containing (*R*)-acetoin and (*S*)-acetoin for calculating the optical purity. The peak area of (*R*)-acetoin (Rt = 2.878 min) was 420.60 and the peak are of (*S*)-acetoin (Rt = 3.023 min) was 15.25. (*R*)-AC, (*R*)-acetoin; (*S*)-AC, (*S*)-acetoin; SS-BD, (*S*,*S*)-2,3-butanediol; RR-BD, (*R*,*R*)-2,3-butanediol; meso-BD, meso-2,3-butanediol; Table S1: Primers used in this study.

## References

[1] Yuan H., Xu Y., Chen Y., Zhan Y., Wei X., Li L., Wang D., He P., Li S., Chen S. (2019). Metabolomics analysis reveals global acetoin stress response of *Bacillus licheniformis*. Metabolomics.

[2] Lu L., Mao Y., Kou M., Cui Z., Jin B., Chang Z., Wang Z., Ma H., Chen T. (2020). Engineering central pathways for industrial-level (3*R*)-acetoin biosynthesis in *Corynebacterium glutamicum*. Microb. Cell Factories.

[3] Cui Z., Wang Z., Zheng M., Chen T. (2021). Advances in biological production of acetoin: A comprehensive overview. Crit. Rev. Biotechnol..

[4] Werpy T., Petersen G. (2004). Top Value added Chemicals from Biomass: Volume I—Results of Screening for Potential Candidates from Sugars and Synthesis Gas.

[5] Zhang L., Liu Q., Ge Y., Li L., Gao C., Xu P., Ma C. (2016). Biotechnological production of acetoin, a bio-based platform chemical, from a lignocellulosic resource by metabolically engineered *Enterobacter cloacae*. Green Chem..

[6] Kandasamy V., Liu J., Dantoft S.H., Solem C., Jensen P.R. (2016). Synthesis of (3*R*)-acetoin and 2,3-butanediol isomers by metabolically engineered *Lactococcus lactis*. Sci. Rep..

[7] Li J., Lu J., Ma Z., Li J., Chen X., Diao M., Xie N. (2022). A green route for high-yield production of tetramethylpyrazine from non-food raw materials. Front. Bioeng. Biotechnol..

[8] Xiao Z., Lu J.R. (2014). Strategies for enhancing fermentative production of acetoin: A review. Biotechnol. Adv..

[9] Meng W., Ma C., Xu P., Gao C. (2022). Biotechnological production of chiral acetoin. Trends Biotechnol..

[10] Jang J.-W., Jung H.-M., Kim D.G., Oh M.-K. (2017). Acetoin production using metabolically engineered *Klebsiella pneumoniae*. Korean Chem. Eng. Res..

[11] Bai F., Dai L., Fan J., Truong N., Rao B., Zhang L., Shen Y. (2015). Engineered *Serratia marcescens* for efficient (3*R*)-acetoin and (2*R*,3*R*)-2,3-butanediol production. J. Ind. Microbiol. Biotechnol..

[12] Yang T., Rao Z., Zhang X., Xu M., Xu Z., Yang S.-T. (2017). Metabolic engineering strategies for acetoin and 2,3-butanediol production: Advances and prospects. Crit. Rev. Biotechnol..

[13] Bae S.-J., Kim S., Hahn J.-S. (2016). Efficient production of acetoin in *Saccharomyces cerevisiae* by disruption of 2,3-butanediol dehydrogenase and expression of NADH oxidase. Sci. Rep..

[14] Sørensen H.P., Mortensen K.K. (2005). Advanced genetic strategies for recombinant protein expression in *Escherichia coli*. J. Biotech..

[15] Xu Y., Chu H., Gao C., Tao F., Zhou Z., Li K., Li L., Ma C., Xu P. (2014). Systematic metabolic engineering of *Escherichia coli* for high-yield production of fuel bio-chemical 2,3-butanediol. Metab. Eng..

[16] Maina S., Prabhu A.A., Vivek N., Vlysidis A., Koutinas A., Kumar V. (2022). Prospects on bio-based 2,3-butanediol and acetoin production: Recent progress and advances. Biotechnol. Adv..

[17] Xu Q., Xie L., Li Y., Lin H., Sun S., Guan X., Hu K., Shen Y., Zhang L. (2015). Metabolic engineering of *Escherichia coli* for efficient production of (3*R*)-acetoin. J. Chem. Technol. Biotechnol..

[18] Xu Y., Xu C., Li X., Sun B., Eldin A.A., Jia Y. (2018). A combinational optimization method for efficient synthesis of tetramethylpyrazine by the recombinant *Escherichia coli*. Biochem. Eng. J..

[19] Guo Z., Zhao X., He Y., Yang T., Gao H., Li G., Chen F., Sun M., Lee J.-K., Zhang L. (2017). Efficient (3*R*)-acetoin production from meso-2, 3-butanediol using a new whole-cell biocatalyst with co-expression of meso-2, 3-butanediol dehydrogenase, NADH oxidase, and *Vitreoscilla* hemoglobin. J. Microbiol. Biotechnol..

[20] Peters D., Ulber R., Sell D. (2007). Raw Materials. White Biotechnology.

[21] Choi G.-W., Moon S.-K., Kang H.-W., Min J., Chung B.-W. (2009). Simultaneous saccharification and fermentation of sludge-containing cassava mash for batch and repeated batch production of bioethanol by *Saccharomyces cerevisiae* CHFY0321. J. Chem. Technol. Biotechnol..

[22] Papong S., Malakul P. (2010). Life-cycle energy and environmental analysis of bioethanol production from cassava in Thailand. Bioresour. Technol..

[23] Wang L., Zhao B., Liu B., Yang C., Yu B., Li Q., Ma C., Xu P., Ma Y. (2010). Efficient production of L-lactic acid from cassava powder by *Lactobacillus rhamnosus*. Bioresour. Technol..

[24] Altaf M., Naveena B.J., Reddy G. (2007). Use of inexpensive nitrogen sources and starch for L(+) lactic acid production in anaerobic submerged fermentation. Bioresour. Technol..

[25] Li Y., Wang L., Ju J., Yu B., Ma Y. (2013). Efficient production of polymer-grade d-lactate by *Sporolactobacillus laevolacticus* DSM442 with agricultural waste cottonseed as the sole nitrogen source. Bioresour. Technol..

[26] Cesselin B., Henry C., Gruss A., Gloux K., Gaudu P., Björkroth J. (2021). Mechanisms of acetoin toxicity and adaptive responses in an acetoin-producing species, *Lactococcus lactis*. Appl. Environ. Microbiol..

[27] Luo Q., Wu J., Wu M. (2014). Enhanced acetoin production by *Bacillus* amyloliquefaciens through improved acetoin tolerance. Process Biochem..

[28] Si H.-M., Zhang F., Wu A.-N., Han R.-Z., Xu G.-C., Ni Y. (2016). DNA microarray of global transcription factor mutant reveals membrane-related proteins involved in n-butanol tolerance in *Escherichia coli*. Biotechnol. Biofuels.

[29] Yuan Y., Bi C., Nicolaou S.A., Zingaro K.A., Ralston M., Papoutsakis E.T. (2014). Overexpression of the *Lactobacillus plantarum* peptidoglycan biosynthesis *murA2* gene increases the tolerance of *Escherichia coli* to alcohols and enhances ethanol production. Appl. Microbiol. Biotechnol..

[30] Kamthan A., Kamthan M., Datta A. (2017). Expression of C-5 sterol desaturase from an edible mushroom in fisson yeast enhances its ethanol and thermotolerance. PLoS ONE.

[31] Fisher M.A., Boyarskiy S., Yamada M.R., Kong N., Bauer S., Tullman-Ercek D. (2014). Enhancing tolerance to short-chain alcohols by engineering the *Escherichia coli* AcrB efflux pump to secrete the non-native substrate n-butanol. ACS Synth. Biol..

[32] Blin K., Pedersen L.E., Weber T., Lee S.Y. (2016). CRISPy-web: An online resource to design sgRNAs for CRISPR applications. Synth. Syst. Biotechnol..

[33] Lv X., Dai L., Bai F., Wang Z., Zhang L., Shen Y. (2016). Metabolic engineering of Serratia marcescens MG1 for enhanced production of (3*R*)-acetoin. Bioresour. Bioprocess..

[34] Hao Z.-k., Li P.-w., Hao Z.-c., Chen L.-f. (2014). Effect of knockouting *frdB* on anaerobic mixed acid fermentation for *Escherichia coli*. China Biotechnol..

[35] Li Z.-y., Wang J., Tian K.-m., Dong Z.-x., Jin P., Liu X.-g., Wang Z.-x. (2018). High-efficiency production of succinic acid from glycerol by metabolically engineered *Escherichia coli*. Sci. Technol. Food Ind..

[36] Cao J., Zhou L., Zhang L., Wang Z., Shi G. (2010). Construction and fermentation of succinate-producing recombinant *Escherichia coli*. Chin. J. Appl. Environ. Biol..

[37] Zingaro K.A., Terry Papoutsakis E. (2013). GroESL overexpression imparts *Escherichia coli* tolerance to *i*-, *n*-, and 2-butanol, 1,2,4-butanetriol and ethanol with complex and unpredictable patterns. Metab. Eng..

[38] Ni Y., Song L., Qian X., Sun Z. (2013). Proteomic analysis of *Pseudomonas putida* reveals an organic solvent tolerance-related gene *mmsB*. PLoS ONE.

[39] Tan Z., Yoon J.M., Nielsen D.R., Shanks J.V., Jarboe L.R. (2016). Membrane engineering via trans unsaturated fatty acids production improves *Escherichia coli* robustness and production of biorenewables. Metab. Eng..

[40] Bui L.M., Lee J.Y., Geraldi A., Rahman Z., Lee J.H., Kim S.C. (2015). Improved *n*-butanol tolerance in *Escherichia coli* by controlling membrane related functions. J. Biotech..

[41] Werbowy O., Werbowy S., Kaczorowski T. (2017). Plasmid stability analysis based on a new theoretical model employing stochastic simulations. PLoS ONE.

[42] Mesa-Pereira B., Rea M.C., Cotter P.D., Hill C., Ross R.P. (2018). Heterologous expression of biopreservative bacteriocins with a view to low cost production. Front. Microbiol..

