# Supplementary material for: Metabolic Engineering of Escherichia coli for High-Level Production of (R)-Acetoin from Low-Cost Raw Materials
Source: Microorganisms. 2023 Jan 13;11(1):203. doi: 10.3390/microorganisms11010203 (PMC9867144; doi:10.3390/microorganisms11010203)
Supplement: Supplementary file 1 [file microorganisms-11-00203-s001.zip › microorganisms-2075643-supplementary.pdf]

Supporting information for:

Metabolic engineering of *Escherichia coli* for high-level pro-duction of (*R*)-acetoin from non-food raw materials cassava

Mengxue Diao [1,§,*], Jing Li [2], Xianrui Chen [1], Ya'nan Shi [3], Bo Yu [3], Jianxiu Li [1] and Nengzhong Xie [1,*]

[1] State Key Laboratory of Non-food Biomass and Enzyme Technology, National Engineering Research Center for Non-food Biorefinery, Guangxi Biomass Engineering Technology Research Center, Guangxi Academy of Sciences, 98 Daling Road, Nanning 530007, China

[2] Life Science and Technology College, State Key Laboratory for Conservation and Utilization of Subtropical Agro-Bioresources, Guangxi University, Nanning 530004, China

[3] CAS Key Laboratory of Microbial Physiological & Metabolic Engineering, Institute of Microbiology, Chinese Academy of Sciences, Beijing 100101, China

[*] Correspondence: mengxuediao@gxas.cn; xienengzhong@gxas.cn

[§] These authors contributed equally to this work.

Content

Figure S1. GC spectrum of fermentation broth containing (*R*)-acetoin and (*S*)-acetoin for calculating the optical purity. The peak area of (*R*)-acetoin (Rt=2.878 min) was 420.60 and the peak are of (*S*)-acetoin (Rt=3.023 min) was 14.20. (*R*)-AC, (*R*)-acetoin; (*S*)-AC, (*S*)-acetoin; SS-BD, (*S*,*S*)-2,3-butanediol; RR-BD, (*R*,*R*)-2,3-butanediol; meso-BD, meso-2,3-butanediol.

Table S1. Primers used in this study

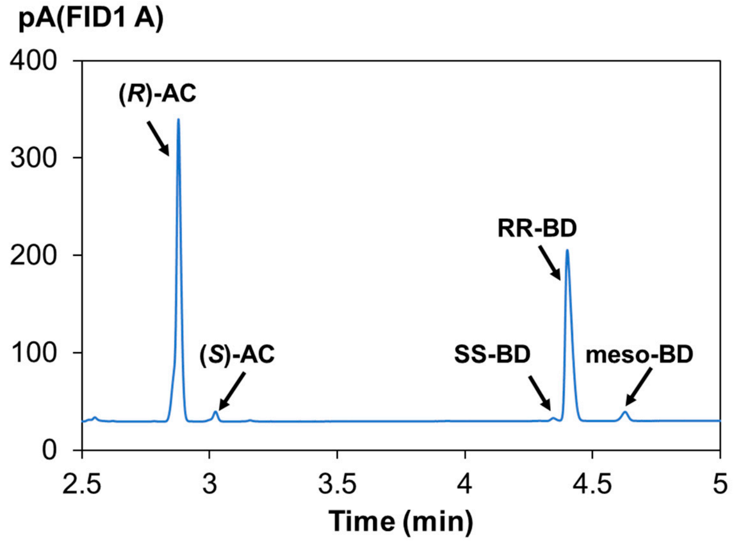

Figure S1. GC spectrum of fermentation broth containing (*R*)-acetoin and (*S*)-acetoin for calculating the optical purity. The peak area of (*R*)-acetoin (Rt=2.878 min) was 420.60 and the peak are of (*S*)-acetoin (Rt=3.023 min) was 15.25. (*R*)-AC, (*R*)-acetoin; (*S*)-AC, (*S*)-acetoin; SS-BD, (*S*,*S*)-2,3-butanediol; RR-BD, (*R*,*R*)-2,3-butanediol; meso-BD, meso-2,3-butanediol.

Table S1. Primers used in this study.

Primer	Sequence 5' – 3'	Application
fabD-F	ATTTCACACAGGAAACAGACCATGGAAATGACGCAATTTGCATTTGTG	Overexpression of *fab*D in pTrc99A
fabD-R	CTCTCATCCGCCAAAACAGCCAAGCTTTTAAAGCTCGAGCGCCGCTGC	
Cti-F	ATTTCACACAGGAAACAGACCATGGAAATGTTGCCAAGACCGTTGGTTG	Overexpression of *Cti* in pTrc99A
Cti-R	CTCTCATCCGCCAAAACAGCCAAGCTTTCAGAGGTTCTCGTAGCGGTTCA	
pTarget-yibT-F	ACTAGTCCCCGTAACGCGATTCCGTCGTTTTAGAGCTAGAAATAGCAAG	Chromosomally gene deletion of *yib*T
pTarget-yibT-R	TAAAACGACGGAATCGCGTTACGGGGACTAGTATTATACCTAGGACTG	
yibT-up-F	GAATGGCATCTTCATCAGCTTC	
yibT-up-R	CCTTTCAAAAGTAAGCAACGTCTGCGATGATATCTCCGTATTACCCCG	
yibT-down-F	GCAGACGTTGCTTACTTTTGAAAGG	
yibT-down-R	GAGCGTCTGAATGCTGGCAAAAC	
pTarget-yghW-F	ACTAGTGAGATCGCCAGCTTAGCGCCGTTTTAGAGCTAGAAATAGCAAG	Chromosomally gene deletion of *ygh*W
pTarget-yghW-R	TAAAACGGCGCTAAGCTGGCGATCTCACTAGTATTATACCTAGGACTG	
yghW-up-F	TATGCAACGCTATCCGCAAGCG	
yghW-up-R	GCAAGATAATACGTATGTTTGACGTTCACCTCATTCGCTATATATTG	
yghW-down-F	TCAAACATACGTATTATCTTGC	
yghW-down-R	GTTATGTTTGTTCTCTTCGACCTG	
yibT::fabD-up-F	GCACCGAGTCGGTGCTTTTTTTGAATTCGATGATATCTCCGTATTACCCCG	Chromosomally gene replacement of *yib*T by *fab*D
yibT::fabD-up-R	GAGCCGGATGATTAATTGTCAAGAATGGCAT	

	CTTCATCAGCTTC
yibT::fabD-F	GAAGCTGATGAAGATGCCATTCTTGACAATT AATCATCCGGCTC
yibT::fabD-R	GTTTTGCCAGCATTCAGACGCTCATTTGTCCT ACTCAGGAGAGCG
yibT::fabD-down-F	CGCTCTCCTGAGTAGGACAAATGAGCGTCTG AATGCTGGCAAAAC
yibT::fabD-down-R	GGGATAACAGGGTAATAGATCTAAGCTTGCA GACGTTGCTTACTTTTGAAAGG
N20-F	ATAATACTAGTACGGCTTTATCGATAAGAAGG TTTTAGAGCTAGAAATAGC
N20-R	GCTATTTCTAGCTCTAAAACCTTCTTATCGATA AAGCCGTACTAGTATTATA
pTarget-F	GTTTTAGAGCTAGAAATAGCAAGTTA
pTarget-R	ACTAGTATTATACCTAGGACTGAGCT
tsf-F	CTTTCAAAAGTAAGCAACGTCTGCTTACGCA GATCACATCTCCGAGGATT
tsf-R	CCGCGCATTCCCGGCGCGGGGTAATACGGAG ATATCATCATGGCCTCAGG
yibt (up)-F	TGCAGGCAACAATGGAACAA
yibt (up)-R	CGCAGCTCTTTTACCAGGGATGCGGTAATTTC AGCCATGTAAGCAGACGT
yibt (down)-F	GATGTCCAAGCAGTCTTAATTATCAAACCATG ATGATATCTCCGTATTACC
yibt (down)-R	GTAATTGCGCGCTGGAATG
sanpianduancexu-F	AACTGAGTGCTGGCGAC
sanpianduancexu-R	GGCAATCCTCATCAGAGAATTGC
